# Denosumab versus zoledronic acid for patients with beta-thalassemia major-induced osteoporosis

**DOI:** 10.1097/MD.0000000000023637

**Published:** 2020-12-18

**Authors:** Mohamed A. Yassin, Mohamed O. Abdel Rahman, Anas A. Hamad, Abdul Razzakh Poil, Mohamed T. Abdelrazek, Radwa M. Hussein, Nancy A. Kassem, Afraa M. Fadul, Sarah A. Elkourashy, Abdulqadir J. Nashwan

**Affiliations:** a Department of Medical Oncology/Hematology, National Centre for Cancer Care & Research (NCCCR); b Department of Laboratory Medicine and Pathology, Biochemistry Section; c Department of Pharmacy, National Centre for Cancer Care & Research (NCCCR); d Department of Rheumatology; e Radiology Department, Hamad Medical Corporation (HMC), Doha, Qatar.

**Keywords:** beta-thalassemia major, denosumab, osteoporosis, zoledronic acid

## Abstract

The main aim of this study is to compare the 2 medications denosumab and zoledronic acid for patients with beta-thalassemia major induced osteoporosis. Patients with B-thalassemia major induced osteoporosis will undergo baseline assessment of the bone densitometry by bone density

(DEXA) scan as a standard of care by the radiology department, then a blood test for bone-specific alkaline phosphatase and type-1 collagen telopeptide will be measured by the chemistry laboratory.

Patients with B-thalassemia major induced osteoporosis, who are 18 years of age or more and willing to participate in the study will be enrolled after consenting by the primary investigator in hematology outpatient clinics. Patients with osteoporosis will receive 1 of the 2 medications; at the end of the year, DEXA scan will be done to compare the response of the 2 medications. The potential risks include drug-related side effects.

The outcome will be measured biochemically by measuring bone-specific alkaline phosphatase and type 1 collagen carboxy telopeptide and radiologically by DEXA scan at baseline and 1 year using *Z* score.

## 1 Introduction

The reported frequency of osteoporosis, even in well-treated Thalassemia patients varies from 13.6% to 50% with an additional 45% affected by osteopenia.^[1]^

Despite the significant improvements in the therapeutic management of beta thalassemia major (BTM) over the past few decades, osteoporosis is still a common finding, even in optimally treated patients.^[2–7]^ The relationships between bone mineral densities (BMD) and several clinical characteristics of hematological markers have been described. Chronic anemia, bone marrow expansion due to ineffective erythropoiesis, iron toxicity, calcium and zinc deficiencies, low vitamin D levels, and endocrine complications have been suggested to contribute to the etiology of bone diseases in BTM. Nevertheless, the complex etiological mechanisms of this heterogeneous osteopathy remain incompletely clarified.^[8–13]^ A complex mechanism controls bone remodeling in human. This mechanism includes the receptor activator of nuclear factor kappa B ligand (RANKL), its natural receptor (RANK), and osteoprotegerin (OPG).^[3,14,15]^ The RANK/RANKL pathway is essential to promote osteoclast formation and activation and prolongs osteoclast survival. OPG acts as a decoy receptor for RANKL and prevents its interaction with RANK thereby inhibiting osteoclast formation, function, and survival. Alteration of the RANK/RANKL/OPG system for increased osteoclastic activity and enhanced osteoblastic dysfunction is proposed as an important mechanism in the etiology of osteoporosis in BTM.^[14,16]^ Hypogonadism, a common finding in BTM, is associated with enhanced RANKL activity.^[17]^ The sex steroid hormones, androgen, and estrogens, via their respective nuclear receptors, regulate BMD in humans and mice. Testosterone is likely to have direct and indirect inhibitory effects on human osteoclast formation and bone resorption.^[18]^ Animal model and cell culture studies suggest a direct inhibitory effect of androgens on the OPG/RANKL cytokines system. In human osteoblastic cells, testosterone and 5 dihydrotestosterone mediate an androgen receptor-induced specific inhibition of OPG messenger ribonucleic acid expression.^[18,19]^ Androgens have also been shown to block RANKL induced osteoclastic formation.^[18,20]^ While RANKL expression was found to be upregulated in osteoblastic cells from androgen receptor-deficient mice.^[21]^ The effect of oestradiol (E2) on osteoclast precursors and osteoclasts seems to be mediated by osteoblastic cells. The inhibitory effect of E2 is associated with the stimulated secretion of OPG by osteoblasts.^[18]^ Previous studies have focused on the characteristics of thalassemic patients with osteoporosis and their response to therapy with bisphosphonates.^[12]^ Because RANK RANKL and OPG play a significant role in bone resorption and seem to be the principal implicated mechanism for the development of osteoporosis in BTM, we will conduct this prospective study to evaluate the anti-RANKL denosumab versus zoledronic acid on TM induced osteoporosis.

## 2 Objectives

The objective of this study is to:

- Evaluate the efficacy and safety of denosumab versus zoledronic acid on the biochemical and radiological parameters of bone mineralization in patients with BTM-induced osteoporosis.

## 3 Trial design

This is a phase-III, Randomized, Comparative, Parallel Assignment, Open-Label clinical study.

## 4 Methods: participants, interventions, and outcomes

### 4.1 Study setting

The study will take place in the National Center for Cancer Care and Research (NCCCR) exclusively where the hematology outpatient clinics will be involved. All patients are diagnosed and/or followed in NCCCR, and all the investigators on this protocol are from NCCCR. The details of the procedure and interventions are mentioned below in the background and methods sections.

### 4.2 Eligibility criteria

Patients with beta-thalassemia major are followed in the Hemoglobinopathy clinic in NCCCR. DEXA scan will be done as part of the routine workup as screening after signing the informed consent; patients with confirmed osteoporosis will be randomized and will continue the study.

The study will not include vulnerable subjects no children, no pregnant ladies, no prisoners Inclusion criteria will be as per the World Health Organization criteria for osteoporosis.^[7]^

*Z*-score will be used (the *Z*-score compares the patient's BMD to an age-matched population). A *Z*-score of –2.0 or lower is considered below the expected range for age. Thus, the presence of *Z*-score values more than 2 standard deviation below the mean should prompt scrutiny for coexisting problems (eg, glucocorticoid therapy or alcoholism) that can contribute to osteoporosis.

- Willing to participate in the study
- Age 18 years old or older
- Eastern Cooperative Oncology Group performance status less than or equal to 2

### 4.3 Exclusion criteria

- Age less than 18 years old
- Not willing to participate in the study
- Vulnerable subjects or Eastern Cooperative Oncology Group Performance Status 3 or 4

### 4.4 Outcomes measures

Primary outcome measure:

1. The number of patients with a 50% or greater reduction in type-1 collagen carboxy telopeptide from the baseline.
  - The number of patients with a 50% or greater reduction in type-1 collagen carboxy telopeptide from the baseline [Time Frame: 12 months].

Secondary outcome measures:

1. The number of patients with a 50% or greater improvement in dual-energy X-ray absorptiometry scan from the baseline.
  - The number of patients with a 50% or greater improvement in dual-energy X-ray absorptiometry scan from the baseline [Time Frame: 12 months].
2. The number of participants with treatment-related adverse events as assessed by Common Terminology Criteria for Adverse Events v4.0 [Time Frame: 12 months].

### 4.5 Participant timeline

The expected time of the trial will be 1 calendar year after ethical approval and will be renewed annually for 5 years.

### 4.6 Sample size and recruitment

This is a pilot study where 40 patients will be recruited, 20 will receive denosumab and 20 will receive zoledronic acid so when a patient with thalassemia major attend their routine visit in NCCCR DEXA scan.

## 5 Methods: data collection, management, and analysis

The DEXA machine will be used in the study is Lunar GE the 2 measurements will be done in the same machine will be requested as the standard of care (value of lumbar spine bone mineral density in gram /cm square and the neck of the hip (Femur). Bone density scan measures bone mineral content (in grams) and bone area (BA, in square centimeters), then calculates “areal” bone mineral density in g/cm^2^) and the World Health Organization criteria for osteoporosis will be applied to patients with osteoporosis (due to complex pathogenesis of beta-thalassemia other factors that could affect bone mineral density will be evaluated like parathyroid hormone, vitamin D, luteinizing hormone, follicle-stimulating hormone, testosterone, iron overload by ferritin, and magnetic resonance imaging). Patients will undergo hormone tests parathyroid hormone, calcium level, and vitamin D level before initiation of the drug administration as the drug can cause Osteomalacia in patients with very low levels of calcium and Vit D; Iron overload will be evaluated for all patients using 2 parameters ferritin and magnetic resonance imaging will be introduced to the study and if they accept they will sign the informed consent and will be randomized for 1 of 2 groups either denosumab twice per year for 1 year or zoledronic acid once per year for 1 year no additional visits apart from regular visits are required, the additional blood test for type 1 collagen carboxy telopeptide and bone-specific alkaline phosphatase will be required as a baseline then every 3 months for 1 year DEXA scan will be done as a baseline and at 30 months; the dose of denosumab will be 60 mg subcutaneous injection every 6 months (twice a year) and zoledronic acid will be 5 mg, intravenous injection once per year. All patients will receive calcium 1000 mg and Vit D 800 IU. All patients will have their assessment by DEXA Scan (lunar GE) throughout the study.

### 5.1 Data management

The responsibilities of the designated study team include project compliance, data collection, abstraction and entry, data reporting, regulatory monitoring, problem resolution and prioritization, and coordination of the activities of the protocol study team.

The collected data for this study will be transferred to a secure database managed by the Hamad Medical Corporation (HMC) IT team (eg, PACS). All data generated in this study will be the property of HMC.

Source documentation will be available to support the computerized patient record. Study personnel will record clinical data in each patient's source documents (ie, the patient's medical record). The study team will maintain adequate and accurate records to enable the conduct of the study to be fully documented and the study data to be subsequently verified. After study closure, the investigators will maintain all source documents, study-related documents, and the data stored in the database used for data collection. Data will be entered throughout the trial as patients are enrolled.

### 5.2 Statistical methods

A well-structured data sheet or data capture form will be designed and created to collect all required data in view of the research study design and objectives. Quality of data (review of completeness, data verification and accuracy, security, and confidentiality of data) will be performed and maintained by lead research investigators. Participant will be reassured regarding the anonymity and confidentiality of the study results and no personal identification will be used in scientific presentations and publications. All patients’ information will be kept confidential and will have access only to the study research investigators and persons authorized by the research ethics committee. Categorical and continuous values will be expressed as frequency (percentage) and mean ± SD or median and interquartile range as appropriate. Descriptive statistics will be used to summarize demographic, bone-specific alkaline phosphatase and type 1 collagen carboxy telopeptide, laboratory and other clinical characteristics of the patients. The primary aim of this study is to evaluate the efficacy and safety of anti-RANKL denosumab versus zoledronic acid on the biochemical and radiological parameters of bone mineralization in patients with BTM induced osteoporosis.

Associations between 2 or more qualitative variables will be assessed using the Chi-square (*χ*^2^) test and Fisher Exact test as appropriate. Quantitative data between the 2 independent groups will be analyzed using unpaired “*t*” or Mann–Whitney *U* test as appropriate. Quantitative data measured at baseline and at 1 year (change from baseline) will be compared using paired *t* and Wilcoxon signed-rank test as appropriate. Differences in bone-specific alkaline phosphatase and type 1 collagen carboxy telopeptide (using *T* and *Z* scores) measured biochemically and radiological at baseline and at every 3 months up to 1 year will be compared using repeated-measures analysis of variance. The results will be presented with the associated 95% confidence interval. The correlation between changes of various biochemical parameters and BMD will be evaluated using Pearson (*r*) or Spearman (*rs*) correlation coefficients. In addition, if needed other appropriate regression analysis (as an exploratory analysis) will be used to assess and quantify the effect of different factors on primary and secondary outcome measures. Pictorial presentations of the key results will be made using appropriate statistical graphs. All *P*-values presented will be 2-tailed, and *P*-values < .05 will be considered as statistically significant. All statistical analyses will be done using statistical packages SPSS 22 (SPSS Inc. Chicago, IL).

## 6 Methods: monitoring

### 6.1 Data monitoring & auditing

The clinical trial master file will be kept along with informed consent from the patients according to the Joint Commission International, Medical Research Center (MRC), and supreme council requirements.

Any side effects or adverse events related to the trial will be reported to the hospital research committee and MRC.

A regular registration report will be generated to monitor patient accrual and completeness of registration data. A routine data quality report will be generated to assess missing data and inconsistencies. Accrual rate and extent and accuracy of evaluations and follow-up will be monitored periodically throughout the study period, and potential problems will be brought to the attention of the study team for discussion and action. Random-template data quality and protocol compliance audits may be conducted by the study team, at a minimum of once per year, or more frequently if indicated. Data safety and monitoring will be conducted according to the HMC-IRB, Ethics, and Data Safety Monitoring Board regulations.

## 7 Ethics and dissemination

### 7.1 Research ethics approval

The study is approved (full-board) by the IRB-MRC at HMC in Qatar (16441/16).

### 7.2 Protocol amendments

None.

### 7.3 Informed consent

The researcher will do screening from patient pool those patients found to be eligible as per inclusion and exclusion criteria will be interviewed to discuss the trial if accepted they will be enrolled in the study the Principal Investigator will do this process the subject will be given free time to decide to participate or not the consenting process will start by screening then interviewing the eligible patient explaining about the trial rationale benefits risks and objectives his or her right to participate or not to participate without being affected and the right to withdraw at any time.

### 7.4 Confidentiality

Patients’ data will be coded and kept in a secure database with a unique username and password to maintain patient confidentiality. Only the authorized research team will be granted access to the patients’ electronic charts and reports.

## Author contributions

**Conceptualization:** Mohamed A. Yassin.

**Methodology:** Mohamed O. Abdel Rahman, Anas A. Hamad, Abdul Razzakh Poil, Mohamed T. Abdelrazek, Radwa M. Hussein, Nancy A. Kassem, Afraa M. Fadul, Sarah A. Elkourashy.

**Project administration:** Abdulqadir J. Nashwan.

**Writing – original draft:** Mohamed A. Yassin, Mohamed O. Abdel Rahman, Anas A. Hamad, Abdul Razzakh Poil, Mohamed T. Abdelrazek, Radwa M. Hussein, Nancy A. Kassem, Afraa M. Fadul, Sarah A. Elkourashy, Abdulqadir J. Nashwan.

**Writing – review and editing:** Mohamed A. Yassin, Mohamed O. Abdel Rahman, Anas A. Hamad, Abdul Razzakh Poil, Mohamed T. Abdelrazek, Radwa M. Hussein, Nancy A. Kassem, Afraa M. Fadul, Sarah A. Elkourashy, Abdulqadir J. Nashwan.
